# PRADA: Portable Reusable Accurate Diagnostics with nanostar Antennas for multiplexed biomarker screening

**DOI:** 10.1002/btm2.10165

**Published:** 2020-05-15

**Authors:** Xiaona Wen, Yu‐Chuan Ou, Holly F. Zarick, Xin Zhang, Anthony B. Hmelo, Quinton J. Victor, Eden P. Paul, Joseph M. Slocik, Rajesh R. Naik, Leon M. Bellan, Eugene C. Lin, Rizia Bardhan

**Affiliations:** ^1^ Department of Chemical and Biomolecular Engineering Vanderbilt University Nashville Tennessee USA; ^2^ Department of Mechanical Engineering Vanderbilt University Nashville Tennessee USA; ^3^ Department of Physics and Astronomy Vanderbilt University Nashville Tennessee USA; ^4^ Materials and Manufacturing Directorate and 711th Human Performance Wing, Air Force Research Laboratory Wright‐Patterson Air Force Base Dayton Ohio USA; ^5^ Department of Chemistry and Biochemistry National Chung Cheng University Chiayi Taiwan; ^6^ Department of Chemical and Biological Engineering Iowa State University Ames Iowa USA; ^7^ Nanovaccine Institute Iowa State University Ames Iowa USA

**Keywords:** biodiagnostic, biosensor, cardiac troponin I, gold nanostars, multiplexing, neuropeptide Y, reusable, surface enhanced Raman

## Abstract

Precise monitoring of specific biomarkers in biological fluids with accurate biodiagnostic sensors is critical for early diagnosis of diseases and subsequent treatment planning. In this work, we demonstrated an innovative biodiagnostic sensor, portable reusable accurate diagnostics with nanostar antennas (PRADA), for multiplexed biomarker detection in small volumes (~50 μl) enabled in a microfluidic platform. Here, PRADA simultaneously detected two biomarkers of myocardial infarction, cardiac troponin I (cTnI), which is well accepted for cardiac disorders, and neuropeptide Y (NPY), which controls cardiac sympathetic drive. In PRADA immunoassay, magnetic beads captured the biomarkers in human serum samples, and gold nanostars (GNSs) “antennas” labeled with peptide biorecognition elements and Raman tags detected the biomarkers via surface‐enhanced Raman spectroscopy (SERS). The peptide‐conjugated GNS‐SERS barcodes were leveraged to achieve high sensitivity, with a limit of detection (LOD) of 0.0055 ng/ml of cTnI, and a LOD of 0.12 ng/ml of NPY comparable with commercially available test kits. The innovation of PRADA was also in the regeneration and reuse of the same sensor chip for ~14 cycles. We validated PRADA by testing cTnI in 11 de‐identified cardiac patient samples of various demographics within a 95% confidence interval and high precision profile. We envision low‐cost PRADA will have tremendous translational impact and be amenable to resource‐limited settings for accurate treatment planning in patients.

## 1 INTRODUCTION

Rapid and accurate detection of disease‐specific biomarkers is imperative for monitoring human health, planning treatment, and responding posttreatment.^1^, ^2^Enzyme‐linked immunosorbent assays (ELISAs) and mass spectrometry are the current clinical standards for detecting and measuring biomarkers in clinical samples. Although these workhorses of clinical laboratories yield accurate diagnostics, long sample preparation times, high operational costs, large sample volumes, and low rates of analysis limit the utility of these techniques for early and rapid detection.^3^ The limitations of current techniques have motivated the development of a broad array of biodiagnostic sensors based on colorimetry, electrochemistry, surface plasmon resonance (SPR), Raman, and fluorescence.^4^, ^5^, ^6^, ^7^, ^8^, ^9^ For clinical applications, biodiagnostic devices must rigorously meet the following functions: (a) multiplexed detection of biomarkers enabling accurate and quantitative bioanalysis at clinically relevant levels; (b) straightforward sample preparation and real‐time readout times; (c) portability and low sample consumption for translation to resource‐limited settings; (d) prolonged reagent shelf life and stability; and (e) reusable to lower diagnostic and analysis costs.^10^, ^11^

**SCHEME 1:**
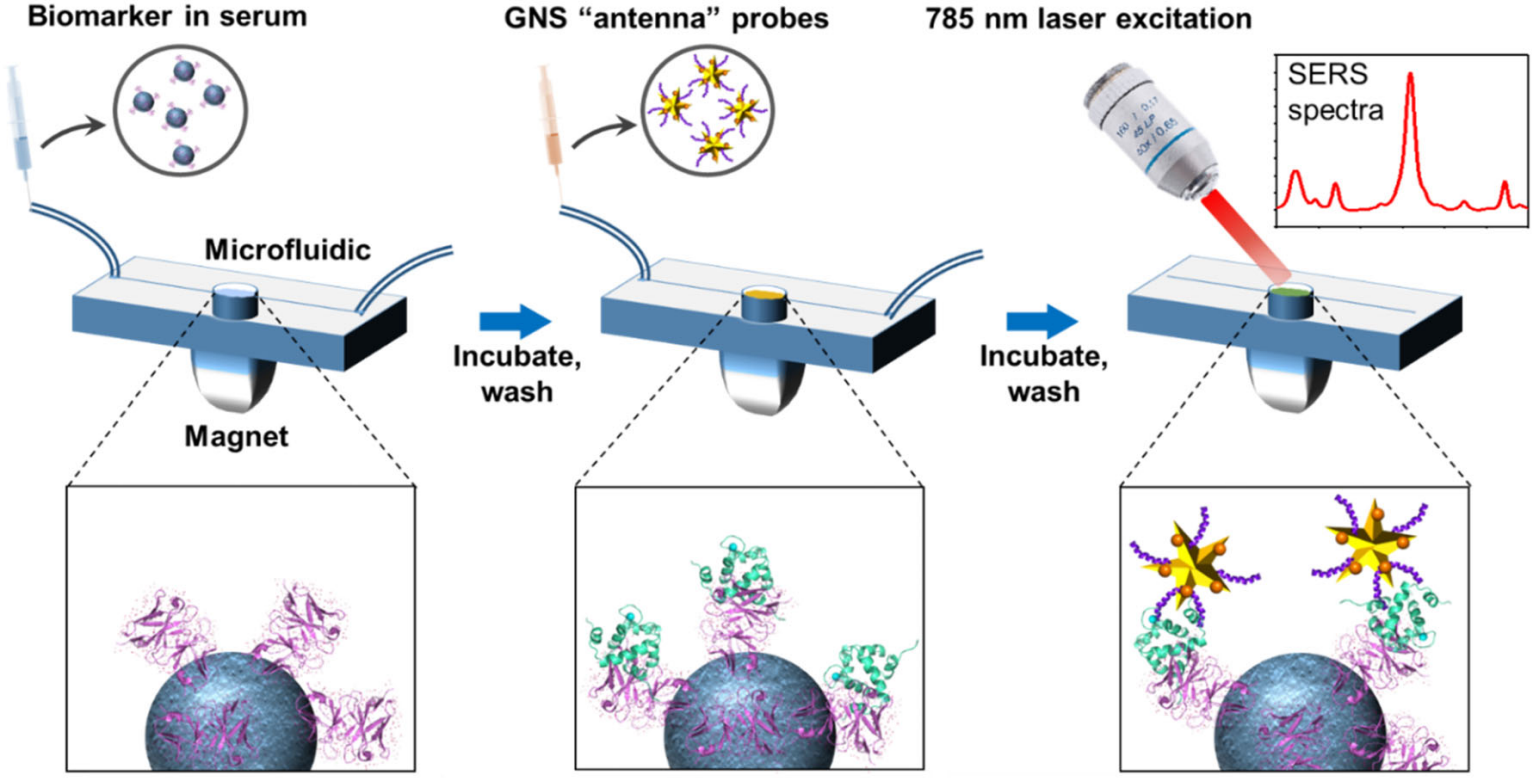
Schematic representation of PRADA. Antibody‐conjugated magnetic beads are incubated with the antigens in the human serum to capture the biomarkers. Raman tags labeled GNSs “antenna” detection probes with peptide BREs then form a sandwich immunocomplex, followed by excitation with 785 nm laser to enable SERS‐based detection. BREs, biorecognition elements; GNSs, gold nanostars; PRADA, portable reusable accurate diagnostics with nanostar antennas; SERS, surface enhanced Raman spectroscopy

In this work, we have designed a new paradigm in diagnostic sensor, PRADA, which synergistically integrates all of these functionalities to allow multiplexed detection of biomarkers in human serum at clinically relevant levels. Portable reusable accurate diagnostics with nanostar antennas (PRADA), is a sandwich immunoassay using polyclonal antibodies (pAbs) functionalized magnetic beads to capture the biomarkers (Scheme 1). Near‐infrared resonant gold nanostars (GNSs) “antennas” labeled with Raman tags and short peptide biorecognition elements (BREs) detect the biomarkers via surface enhanced Raman spectroscopy (SERS). The immunoassay is assembled in a microfluidic device to allow low sample volumes, minimize the assay time, facilitate multiplexed detection, and enable reusability of PRADA. The seamless integration of each of the components of PRADA into a single functional platform allowing a portable and affordable multiplexed biodiagnostic is unprecedented. SERS is a promising immunodetection technique due to its exceptional sensitivity, specificity, and multiplexing ability with minimal spectral overlap between various reporter molecules.^12^, ^13^, ^14^, ^15^, ^16^ The antenna‐like behavior of GNSs is attributable to their unique geometry, where their core acts as an antenna and absorbs near‐infrared light and their branches behave as emitters to localize the absorbed light at the tips to generate intense electric fields.^17^, ^18^, ^19^ We have shown that the near‐field electromagnetic radiation generated at the protrusions of GNSs gives rise to a > 10^9^ enhancement in SERS signal, resulting in ultrasensitive detection in vitro, in vivo, and in biosensors.^20^, ^21^, ^22^, ^23^, ^24^

Here, we demonstrated multiplexed detection of two biomarkers of myocardial infarction including cardiac troponin I (cTnI) and neuropeptide Y (NPY) with PRADA with high sensitivity and specificity in patient sera. cTnI is a well‐accepted serum biomarker of cardiac arrest, stress, and ischemic stroke.^25^, ^26^ cTnI is routinely assessed in patient samples in clinical laboratories to detect myocardial damage, with a clinical range for at‐risk patients of >0.03 ng/ml.^27^ NPY is a sympathetic cotransmitter and critical to cardiovascular homeostasis including cardiac remodeling and angiogenesis.^28^, ^29^ It has been correlated to stress, anxiety, and posttraumatic stress disorder^30^, ^31^ at a clinically relevant level of ≤1.5 ng/ml.^32^ Our results showed that PRADA achieved highly sensitive detection of both biomarkers of acute myocardial infarction ideal for risk stratification. The high sensitivity and specificity of PRADA were leveraged by the peptide BREs conjugated to GNS‐SERS barcodes. Short peptides represent an attractive alternative to monoclonal antibodies (mAbs) due to their low cost, long shelf life, and stability, and their small size enables high sensitivity in PRADA.^33^, ^34^ We also demonstrated that PRADA was reusable where the microfluidic device can be regenerated for multiple cycles. We envision that PRADA will be ultimately useful in resource‐limited settings, where a low‐cost, reusable, and user‐friendly point‐of‐care is necessary for patient sample analysis given that affordable portable Raman spectrometers are now readily available.

## 2 RESULTS AND DISCUSSION

### 2.1 Synthesis and characterization of PRADA

The design of PRADA (Scheme 1) includes pAbs functionalized magnetic beads that were assembled onto a passivated microfluidic device via a magnet to form a uniform layer. These capture probes were then incubated with human serum to capture the relevant biomarkers through the antibody–antigen interactions. Next, GNSs labeled with Raman tags (GNS‐SERS barcodes) and small peptide BREs were introduced which bound to different sites on the biomarkers, completing the sandwich immunocomplex. This assay was followed by SERS measurements with a Raman setup equipped with a 785 nm continuous‐wave laser and analyzed for quantification of the antigens present in serum. Here, we first showed the individual detection of cTnI and NPY followed by multiplexed detection of both biomarkers simultaneously.

**FIGURE 1:**
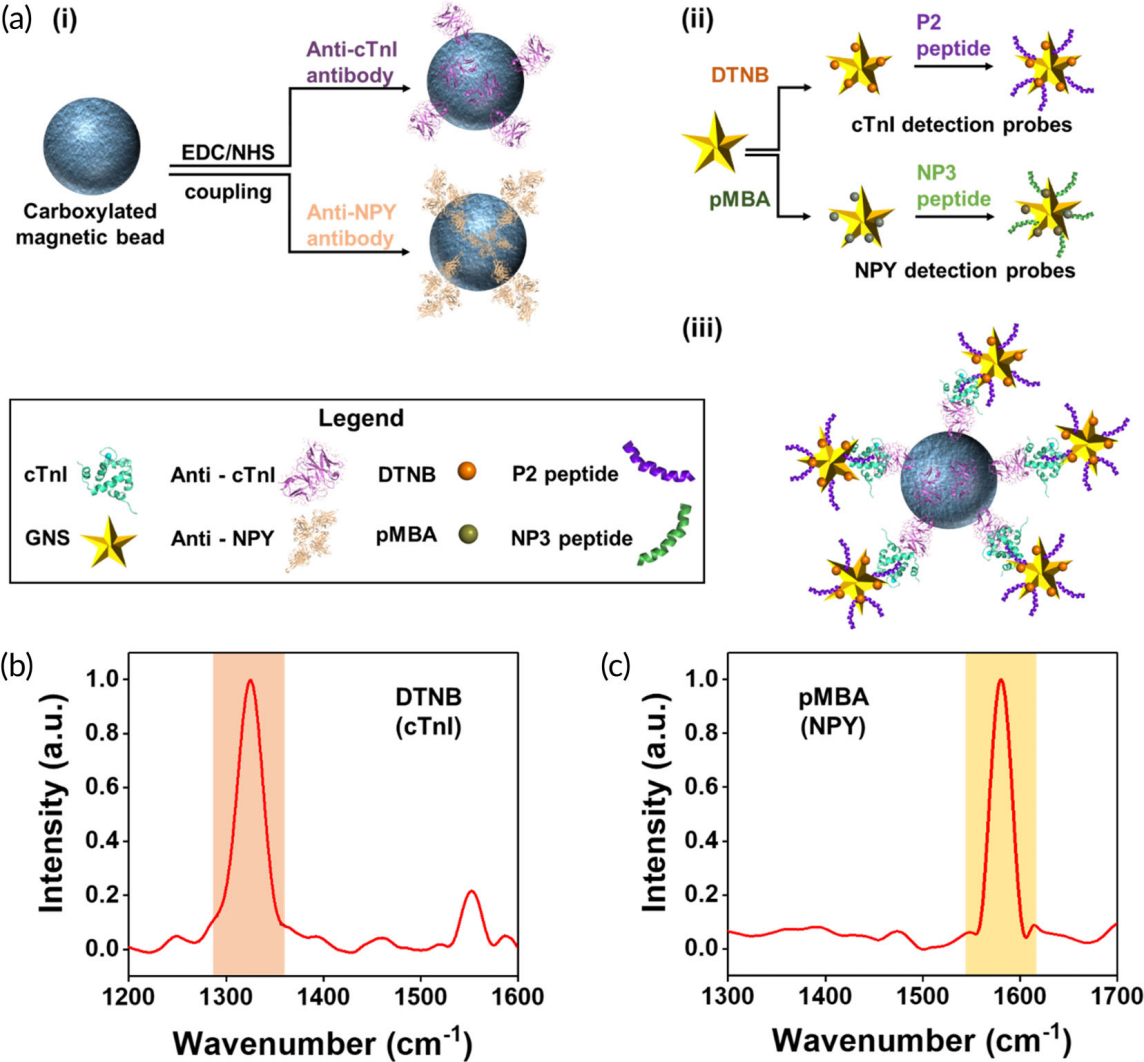
Fabrication of PRADA. (a) Schematic of the synthesis of capture and detection probes. (i) Magnetic beads functionalized with pAbs as capture probes. (ii) GNSs conjugated with SERS barcodes and peptide BREs as detection probes. (iii) The representative complete immunocomplex formed by capture probes, target antigens, and detection probes. (b, c) Normalized Raman spectra of GNSs functionalized with DTNB (1,325 cm^−1^) and pMBA (1,580 cm^−1^) for cTnI and NPY detection, respectively; the signature peaks are highlighted. BREs, biorecognition elements; GNSs, gold nanostars; pAbs, polyclonal antibodies; PRADA, portable reusable accurate diagnostics with nanostar antennas; SERS, surface enhanced Raman spectroscopy

**FIGURE 2:**
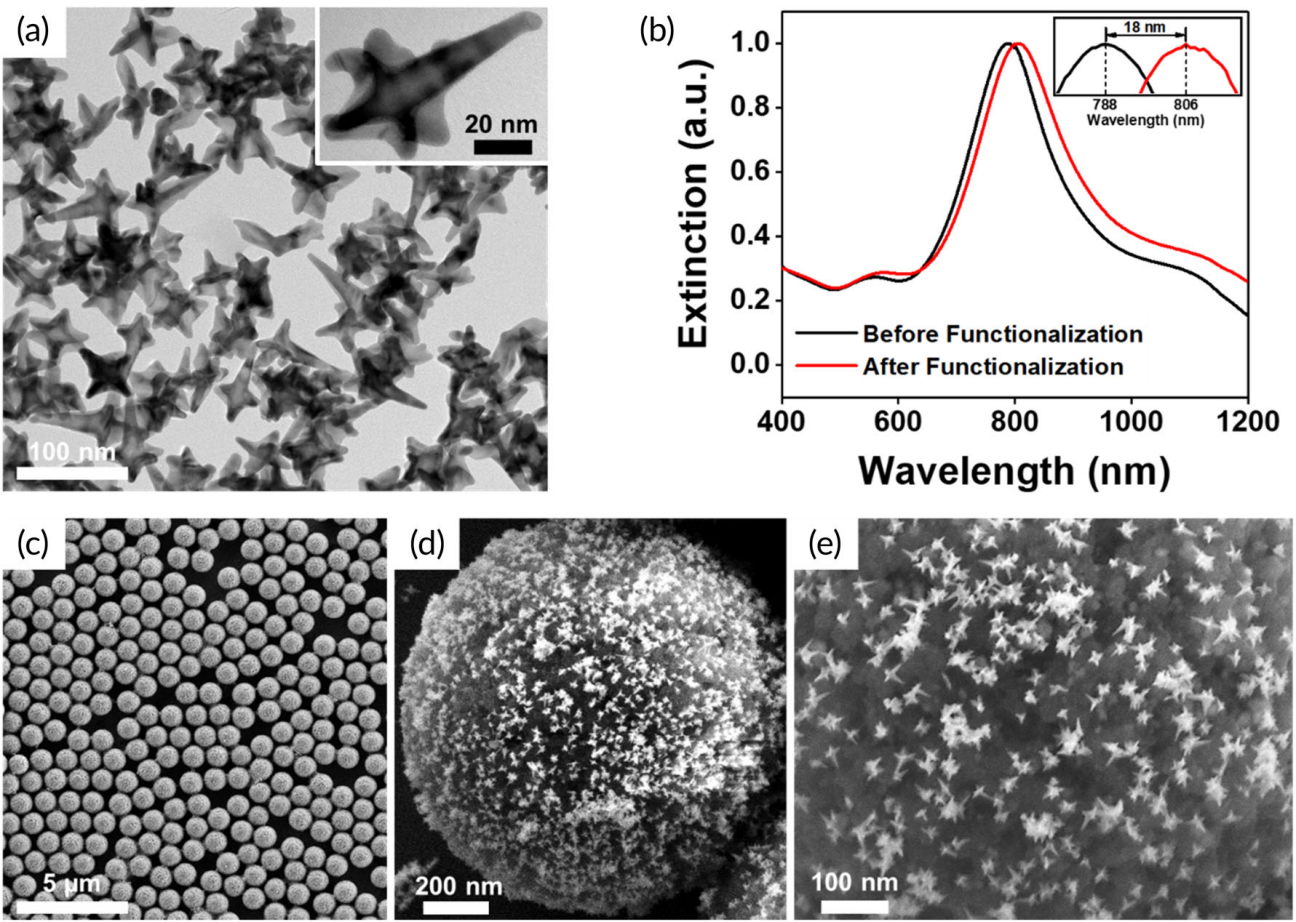
Characterization of PRADA. (a) Transmission electron micrograph of GNSs showing their anisotropic morphology. (b) Extinction spectra of bare GNSs and GNSs functionalized with SERS barcodes and peptide BREs. (c–e) SEM images of complete immunocomplexes at various magnifications with magnetic beads capture probes and GNS‐SERS barcodes detection probes. BREs, biorecognition elements; GNSs, gold nanostars; PRADA, portable reusable accurate diagnostics with nanostar antennas; SEM, scanning electron micrograph; SERS, surface enhanced Raman spectroscopy

The sensitivity and specificity of PRADA are governed by the controlled synthesis of the capture and detection probes (Figure 1a). Here, the capture probes were prepared by activating carboxylic acid‐functionalized magnetic beads via 1‐ethyl‐3‐(3‐dimethylaminopropyl) carbodiimide (EDC) and N‐hydroxysulfosuccnimide (NHS) coupling, and subsequent functionalization with anti‐cTnI or anti‐NPY pAbs. Functionalized capture probes were then incubated with human serum spiked with cTnI or NPY antigens where biomarkers were captured via antibody–antigen binding. The sandwich immunocomplex was completed with GNS‐SERS barcodes covalently conjugated with peptide BREs. The detection of cTnI was enabled with GNSs labeled with 5,5‐dithio‐bis‐(2‐nitrobenzoic acid) (DTNB) Raman tags and P2 peptides (Figure 1b), and detection of NPY was facilitated with GNSs labeled with para‐mercaptobenzoic acid (pMBA) Raman tags and NP3 peptides (Figure 1c). Here, GNSs with 50–70 nm tip‐to‐tip dimension (Figure 2a) were synthesized with a biological buffer, 2‐[4‐(2‐hydroxyethyl)piperazin‐1‐yl]ethanesulfonic acid (HEPES), as described in our previously published procedures.^18^, ^19^, ^20^, ^21^ Further, DTNB and pMBA are ideal Raman tags for this platform because they are covalently linked to the GNS surfaces via a thiol group, enabling SERS signal amplification via both electromagnetic and chemical enhancements. The dominant Raman peaks at 1,325 cm^−1^ (symmetric stretching mode of the nitro group of DTNB) and 1,580 cm^−1^ (ring stretching mode of pMBA) also do not overlap enabling multiplexed detection of both biomarkers.

In our platform, the peptide BRE plays a critical role in the biomarker detection. The P2 peptides (‐WQIAYNEHQWQGGGC‐), computationally evolved from a phage display peptide, had nanomolar binding affinity to cTnI.^35^ The bioconjugation of P2 peptides to GNSs was achieved via Au‐S linkage by introducing a cysteine residue at the C‐terminus of the peptide. A tri‐glycine spacer domain was inserted between the C‐terminal cysteine and the P2 peptide to extend the binding domain away from the gold surface. The NPY binding peptide, NP3 (‐FPNWSLRPMNQMGGGC‐), was also identified from a phage display peptide library.^36^ The short peptides bind to different regions of antigens without competing with the target sites of antibodies on the capture probes. These dodecapeptides have an average size of 2–3 nm obtained by molecular modeling calculations; this size is the molecular length of a linear, unconstrained, and extended dodecameric peptide.^37^ The peptide functionalized and constrained on a nanoparticle surface is likely to have a smaller size. The peptide size is significantly smaller than mAbs (~10 nm) which facilitated high sensitivity of PRADA by enabling the nanostars to maintain their orientation with respect to the antigen receptor with minimal steric hindrance. Antibodies are typically attached to gold nanoparticle surfaces via long chain linkers, which often compromise their orientation and consequently binding efficacy, lowering overall sensitivity.^34^ Of note, we chose to use P2 peptide instead of anti‐cTnI antibody as the peptide was evolved to bind residues 114‐141 of troponin with high affinity whereas anti‐cTnI antibody bound to the N‐terminus region of full‐length troponin. The binding affinity of P2 peptide was confirmed in our previous work by measuring the dissociation constants (K_D_) of the P2 peptide or mAb in the presence of 114‐141 troponin fragment or full‐length troponin.^35^ The K_D_ of the peptide remained constant independent of troponin target (fragment or full length), while the K_D_ of the antibody was 100‐fold lowered using the troponin fragment lacking the N‐terminus binding region. These results confirmed that the peptide and antibody binding domains were nonoverlapping. But the binding affinity of the peptide and antibody for full length troponin was very similar (0.27 vs. 0.12 nM), as measured by SPR.^35^, ^38^

After biofunctionalization of GNSs with peptide BREs and SERS barcodes, a ~ 18 nm red shift in the plasmon resonance (Figure 2b) was observed attributed to an increase in hydrodynamic size and change in refractive index of the medium. Scanning electron micrograph (SEM) images confirmed the successful synthesis of the complete immunocomplex (Figures 2c–e and S1) where GNS‐SERS barcodes retained their morphology after complexing with functionalized magnetic beads. Note: SERS measurements were only acquired from samples where the magnetic beads formed a uniform monolayer aided with a magnet (Figure S2a,b). Samples with multilayers of the complete immunocomplex or aggregated GNSs were avoided to minimize hot‐spot formation and variability in the measurements (Figure S2c–f).

### 2.2 Biomarker detection and reusability

**FIGURE 3:**
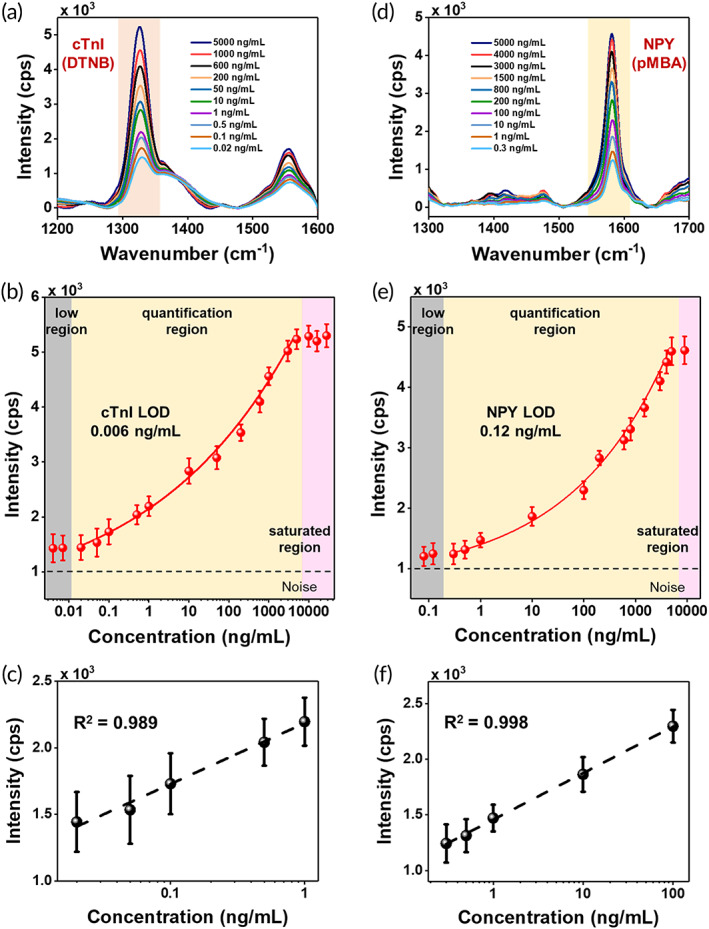
cTnI and NPY detection with PRADA. (a) Raman spectra of cTnI in human serum and (b) SERS intensity at the characteristic DTNB peak (1,325 cm^−1^) as a function of cTnI concentrations. The low region (gray box) was where the concentrations were below the detection limit; quantification region (yellow box) was used to determine LOD with a 4PL function fit; and saturated region (pink box) was where high density of analytes saturated PRADA. (c) Linear fit of the DTNB peak at low concentrations of cTnI in the range of 0.02 to 1 ng/ml. (d) Raman spectra of NPY in human serum by monitoring the pMBA peak at 1580 cm^−1^. (e) SERS intensity at the characteristic pMBA peak as a function of NPY concentrations. (f) Linear fit of the pMBA peak at low concentrations of NPY ranging from 0.3 to 100 ng/ml. Error bars indicate the standard deviations from at least five measurements. A base 10 logarithmic scale was used for x‐axis. 4PL, four‐parameter logistic; LOD, limit of detection; PRADA, portable reusable accurate diagnostics with nanostar antennas; SERS, surface enhanced Raman spectroscopy

We first demonstrated the feasibility of PRADA in detection of single biomarkers in human serum in a microfluidic device. We chose a simple and low‐cost microfluidic design with an inlet and outlet, and a sample chamber for incubation of samples, mixing, and evaluation of biomarkers (Scheme 1). The magnetic bead capture probes, which are uniformly distributed in the entire sample chamber (Figure S2a,b), also aid in mixing with the peptide‐coated GNS‐SERS barcode detection probes by placing the microfluidic devices on a stir plate. Therefore, the design of microfluidic chips with multiple mixing channels is unnecessary here as such complex devices are both time and labor intensive, and cost prohibitive.^39^, ^40^ We chose to measure the accuracy of PRADA in commercially available de‐identified human patient serum (Discovery Life Sciences Inc.) to recapitulate clinical diagnostics where biomarkers of interest compete with other serum constituents to be captured by the magnetic beads. Human serum contains ~4,000 metabolites,^41^, ^42^ which would compete to bind to the targeted sites. Here, different concentrations of cTnI or NPY were spiked into human serum and followed by monitoring the change in intensity of the signature peaks of the GNS‐SERS barcodes bound to cTnI or NPY antigen via the peptide BREs. The cTnI baseline of the purchased serum was 0.015 ng/ml, whereas the amount of NPY in the serum was not provided by Discovery Life Science. However, a blank Raman signal of the serum was obtained in the absence of antigens (with the capture and detection probes). Minimal interference effects were observed in the blank control, which suggested minimal NPY baseline. Each sample was prepared with three replicates, and the sensitivity and specificity of PRADA were quantitatively evaluated. At least 300 spectra obtained from different locations per replicate of sample were used for quantitative analysis. Spectra were background subtracted, averaged, and smoothed using a Savitzky–Golay filter.^43^ The representative Raman spectra of immunocomplexes for various concentrations of cTnI were shown in Figure 3a. The relative SERS intensity of DTNB at 1325 cm^−1^ was used for quantitative evaluation of cTnI concentrations. The corresponding sensitivity curve obtained from the SERS measurements was fitted using the four‐parameter logistic (4PL) function (Table S1) showing that the Raman intensity increased in a logarithmic manner with increasing concentrations of cTnI in the range of 0.02 to 5,000 ng/ml (Figure 3b). This 4PL function has been shown previously to have a robust fit to plasmonic and SERS‐based biosensors.^5^, ^44^ However, the SERS intensity at 1325 cm^−1^ was linear at low concentrations of cTnI in the quantification region (Figure 3c). The LOD of cTnI was estimated to be 0.0055 ng/ml from this fit; all parameters for LOD calculation are provided in Table S2. Our assay is also clinically relevant because patients diagnosed with myocardial infarction typically have a cTnI concentration of >0.03 ng/ml.^27^ Of note, the low region indicated the concentrations below the LOD of PRADA and the saturated region represented where the sandwich immunocomplex was oversaturated and unable to distinguish differences in such high concentrations.^45^

We followed a similar approach in the utility of PRADA to detect NPY spiked in de‐identified human serum. The signature Raman peak of pMBA at 1,580 cm^−1^ was monitored (Figure 3d). The quantification region also showed a logarithmic increase with NPY concentrations ranging from 0.3 to 5,000 ng/ml (Figure 3e), whereas a linear correlation was found in the range of 0.3 to 100 ng/ml (Figure 3f). The LOD of NPY was calculated to be 0.12 ng/ml (Table S2). The clinical level of NPY of at‐risk patients with high level of stress and anxiety is ≤1.5 ng/ml and lower concentrations are desired for risk prediction.^32^ These results demonstrated that PRADA is a versatile platform for quantitative analysis of biomarkers in human biofluids with high sensitivity and specificity.

**FIGURE 4:**
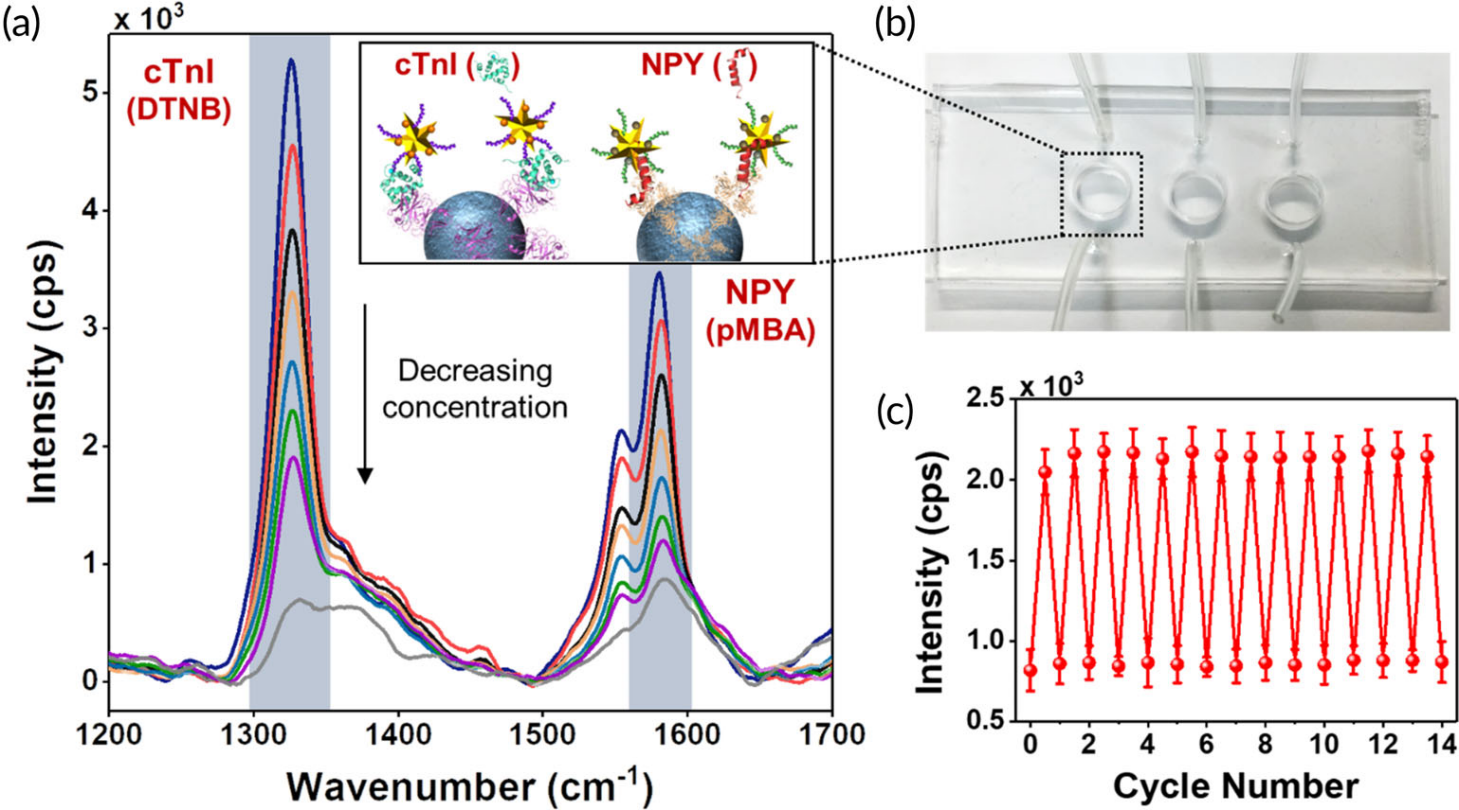
Multiplexing and reusability with PRADA. (a) Multiplexed detection of both cTnI and NPY in a three‐well microfluidic device. The concentrations measured in ng/ml for cTnI/NPY are 3000/1500 (blue), 1000/600 (red), 400/200 (black), 200/100 (orange), 5/10 (light blue), 0.5/1 (green), 0.05/0.3 (purple), and control (gray). The inset is a schematic representation of multiplexed detection of both biomarkers. (b) Image of a microfluidic device utilized in the experiments. (c) Demonstration of reusability of PRADA where the same microfluidic chip was reused 14 times after washing and regenerating. Here, 0.1 ng/ml of cTnI was detected at the DTNB Raman peak with minimal signal loss. Error bars indicate the standard deviations from at least five measurements. PRADA, portable reusable accurate diagnostics with nanostar antennas

Next, we demonstrated that microfluidics‐based PRADA enabled accurate multiplexed detection of biomarkers in serum. Multiplexed bioanalysis in a single sample is of significant interest to predict the complex phenotype of myocardial infarction, which often results in false prognosis.^46^ Here, the narrow spectral features of SERS allowed multiplexed detection offering high sensitivity and minimum overlap between corresponding Raman tags. We simultaneously detected cTnI and NPY (Figure 4a) by using a 1:1 mixture of magnetic beads conjugated with either anti‐cTnI pAbs or anti‐NPY pAbs, which served as the capture probes in a multiwell microfluidic device (Figure 4b). A multiwell device is particularly relevant for field‐use or in resource‐limited settings where several patient samples can be analyzed at the same time to determine the status of multiple biomarkers. Afterwards, serum samples with no antigens (control), 1:1 mixture of cTnI and NPY at various concentrations (see Figure 4 caption) were incubated with the capture probes. After removing unbound antigens with a washing step, 1:1 mixture of detection probes targeting cTnI (GNS‐P2‐DTNB) and NPY (GNS‐NP3‐pMBA) were incubated. Multiplexed detection was achieved with PRADA where clear peaks of DTNB (1,325 cm^−1^) and pMBA (1,580 cm^−1^) were observable with minimal nonspecific binding for the no antigen control. Additionally, the Raman signal of both biomarkers intensified with the increase in biomarkers' concentration.

We then demonstrated that PRADA could be reused over multiple cycles by simply washing and regenerating the microfluidic devices. We used the same microfluidic device to detect 0.1 ng/ml of cTnI for 14 cycles by repeated washing and reusing (Figure 4c). The reusability of PRADA was leveraged with the magnetic beads, as removal of the magnet allowed us to wash off the entire assay and regenerate the microfluidic sensor chip. Our regeneration approach has several merits. First, PRADA had minimal signal loss after multiple cycles, which outperformed chemical regeneration approaches that have been reported to have ~40% signal loss during each cycle.^47^ In chemical regeneration, low pH glycine buffer or detergent solutions are introduced to detach the antigens from antibodies, enabling reusability of the sensor chip with the same set of antibodies between samples.^48^ However, chemical regeneration is ineffective when using patient biofluids due to the presence of proteases and bacteria that can degrade these antibodies.^49^ Low signal loss after multiple cycles emphasized the strength of PRADA and our magnetic regeneration approach. Second, prior to regeneration, the microbead/antigen/nanostar immunocomplex representing each patient sample can be archived (by removing the magnet) for future analysis. Sample archival is possible due to the high stability of the peptide‐coated GNS‐SERS barcodes as they do not photobleach and are amenable to long‐term storage.^24^ These results demonstrated that PRADA is a robust multiuse platform that allows diagnosis of multiple biomarkers of interest within minutes and has the potential to analyze patient samples in biofluids with high accuracy and specificity.

### 2.3 Evaluation of PRADA for clinical samples

**FIGURE 5:**
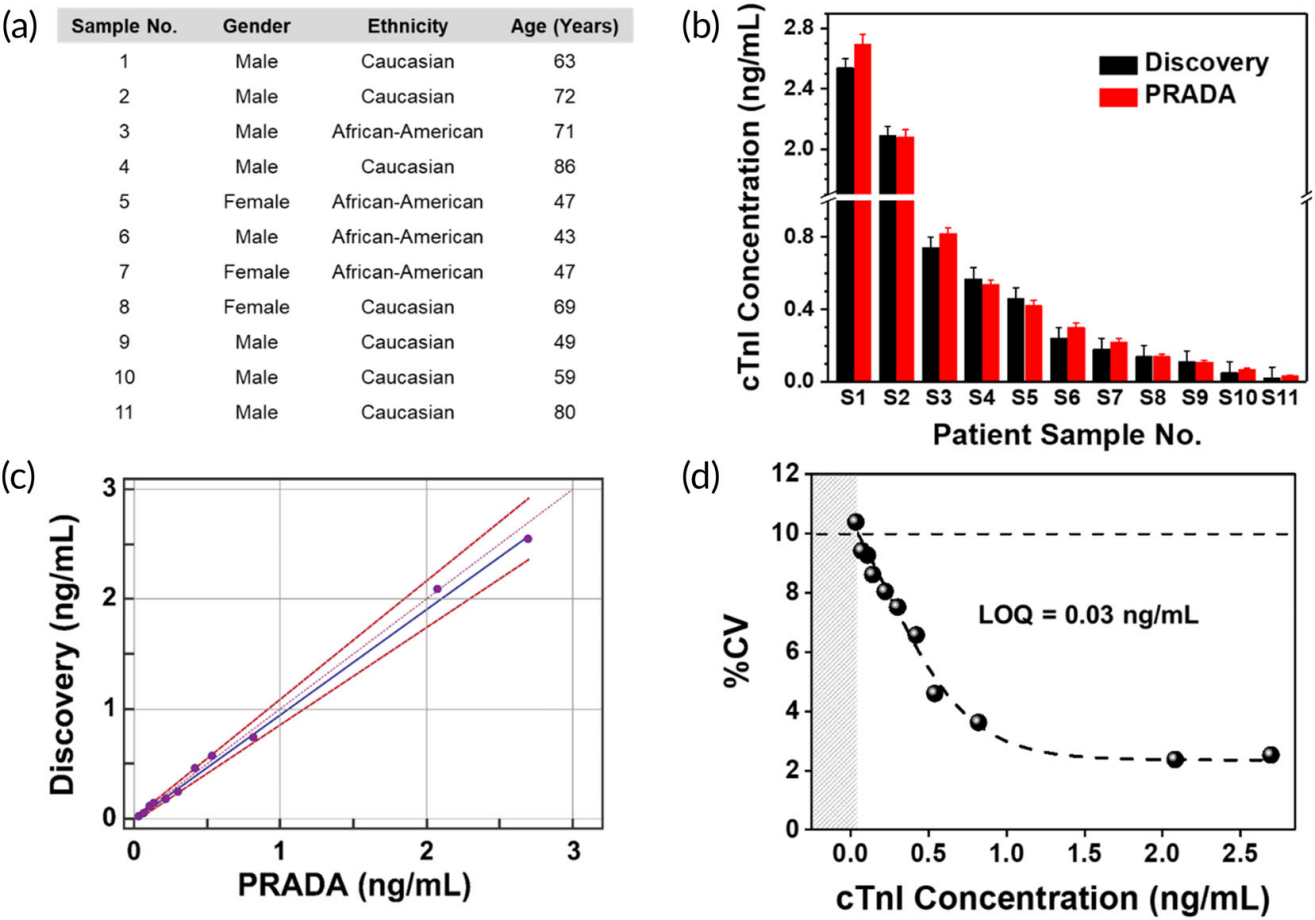
PRADA for cardiac patient sample analysis. (a) Demographics of 11 patient samples purchased from Discovery Life Sciences including their gender, race, and age, and the cTnI levels. (b) Comparison of cTnI determined with PRADA and those obtained from Discovery Life Sciences measured using the ABBOTT ARCHITECT chemiluminescence assay system. The standard errors in Discovery data were <0.06 ng/ml. Error bars in SERS data indicate the standard deviations from at least five measurements. (c) Passing‐Bablok regression analysis between PRADA and Discovery Life Sciences to determine accuracy of PRADA. (d) %CV corresponding to mean cTnI concentrations for the 11 patient samples using PRADA where the 10% CV level is indicated with a dotted line achieving a LOQ of ~0.03 ng/ml. CV, coefficient variation; LOQ, limit of quantification; PRADA, portable reusable accurate diagnostics with nanostar antennas; SERS, surface enhanced Raman spectroscopy

We then demonstrated the translational impact of PRADA by validating our approach in evaluating serum from 11 de‐identified cardiac patients with varying levels of cTnI. These serums were purchased from the open biobank Discovery Life Sciences. The NPY values were not provided for these samples. A three‐well microfluidic device was used to enable multiple patient sample analysis simultaneously. The PRADA assay was performed similar to described above where capture probes for cTnI were introduced in microfluidic wells followed by introducing the patient serum, and then followed by the peptide‐coated GNS‐SERS barcodes. Multiple washing steps were executed to ensure high accuracy of PRADA. Relevant patient information including gender, race, and age is shown in Figure 5a. Our effort was to demonstrate that PRADA is applicable to a wide range of patient demographics including gender, ethnicity, and age. SERS spectra of the patient samples were measured in triplicates (Figure S3) to determine the cTnI levels. PRADA levels were then compared with those provided by Discovery Life Sciences measured using the ABBOTT ARCHITECT chemiluminescence assay system (Figure 5b). The manufacturer provided the standard error in their data to be <0.06 ng/ml where the level consistent with acute myocardial infarction was ≥0.5 ng/ml based on their samples. Our results showed regardless of the cTnI concentration and patient demographics, PRADA achieved high accuracy in serum analysis. Further, Passing‐Bablok regression analysis was performed to estimate the variation and systematic bias between cTnI concentrations obtained with PRADA in patient samples and the values from Discovery Life Sciences (Figure 5c). Regression analysis indicated good conformity between the two assays as the scattered points (purple) and associated regressions (blue) of the data were within the 95% confidence intervals (CIs) which include the intercept and slope of 0 and 1, respectively (Table S3). The results showed that all values obtained with PRADA were clinically valid, and within the acceptable range.

We calculated a precision profile, which determined the limit of quantification (LOQ) of PRADA in analyzing patient samples and ensuring that these results matched with our sensitivity curve shown in Figure 3b,c. Here, the LOQ is defined as the lowest concentration of cTnI that can be reliably detected with a coefficient variation (CV) less than or equal to 10%.^50^ By definition LOQ can be equal to or higher than the LOD but not lower, as LOD provides an estimate of bias and imprecision at very low analyte concentrations. We obtained the mean cTnI concentrations with PRADA for the 11 patient sera (measured in triplicates) and calculated the %CV for each sample (Figure 5d). A curve was fitted through the plot of %CV as a function of cTnI concentration, and the LOQ of PRADA was determined to be 0.03 ng/ml corresponding to the 10% CV level of the curve. The data suggest that PRADA will allow quantitative analysis of cTnI in patient sera at ≥0.03 ng/ml with high accuracy (right of the gray region). Our reported LOD and LOQ are lower than many troponin immunoassays published in the literature, and comparable to commercial assays (Table S4). Of note, multiplexing is not offered by many of these commercial assays. However, our LOQ was limited by the availability of patient samples from Discovery Life Sciences where the lowest concentration of cTnI that was available for purchase was >0.01 ng/ml. We were also limited by the small patient population we evaluated here (11); most commercially available assays examine >1,000 patients to establish their quantification range^27^, ^51^ where patients with no risk of myocardial infarction have ≪0.01 ng/ml. We expect the LOQ of PRADA will be significantly improved in a future cohort study where more patients will be recruited to exemplify the translational impact of this platform.

## 3 CONCLUSIONS

In summary, this study presents an innovative biodiagnostic platform, PRADA, demonstrating multiplexed detection of two biomarkers of myocardial infarction, cTnI and NPY, in a simple microfluidic device. We achieved a LOD of 0.0055 ng/ml for cTnI and a LOD of 0.12 ng/ml for NPY in patient serum. We showed that PRADA can be regenerated and reused where the same microfluidic chip can be recycled for multiple cycles with minimal signal loss between cycles. Reusability of PRADA also allowed archiving samples for future bioanalysis. Finally, we validated the clinical significance of PRADA by evaluating cTnI in cardiac patient serum of various demographics and achieved a LOQ of ≥0.03 ng/ml at 10% CV which is lower than many troponin immunoassays published in the literature, and comparable to commercial assays. Whereas in this proof‐of‐concept study, we demonstrated the multiplexing of two biomarkers in human serum, our future work focuses on the utility of PRADA to detect >10 biomarkers in patient samples enabled by the narrow spectral features of SERS. Further, PRADA can be translated to other biomarkers beyond those probed here, as identification of peptides that exhibit high binding affinities to various targets has already been pursued by many commercial sources that routinely generate a number of different peptides. Although this approach is limited by biomarker targets wherein the antigen structure is known and the location of a binding site on the target molecule can be predicted, we envision that PRADA will ultimately enable a precise scoring system to determine patient outcome. PRADA score can then be integrated with artificial intelligence interfaces as well as smart phones for cost‐effective health monitoring.^52^, ^53^

## 4 MATERIALS AND METHODS

### 4.1 Materials

Carboxylated magnetic beads, 1‐ethyl‐3‐(3‐dimethylaminopropyl) carbodiimide (EDC), N‐hydroxysulfosuccnimide (NHS), 2‐(4‐morpholino)ethane sulfonic acid (MES), tris(hydroxymethyl)aminomethane (Tris base), acetone and microscope glass slides were purchased from ThermoFisher Scientific. Gold (III) chloride trihydrate (HAuCl_4_), (4‐(2‐hydroxyethyl)‐1‐piperazineethanesulfonic acid) (HEPES), Sylgard 184, phosphate‐buffered saline (PBS), ethanol, and trichloro(phenyl)silane (TCPS) were purchased from Sigma‐Aldrich. The Milli‐Q water (18 MΩ) was obtained from a Milli‐Q Direct‐Q 3UV system. Anti‐cTnI (ab47003) and anti‐NPY antibodies (ab30914) were purchased from Abcam. Raman tags, 5,5‐dithio‐bis‐(2‐nitrobenzoic acid) (DTNB) and para‐mercaptobenzoic acid (pMBA), were purchased from TCI America. P2 (‐WQIAYNEHQWQGGGC‐) and NP3 (‐FPNWSLRPMNQMGGGC‐) peptides were purchased from Genscript. Methoxy poly(ethylene glycol)‐silane (mPEG‐silane, MW 5000) was purchased from Laysan Bio. Patient samples were purchased from Discovery Life Science.

### 4.2 Instrumentation

The plasmon resonance of bare and functionalized GNSs was measured with a Varian Cary 5,000 UV–Vis NIR spectrophotometer. The size and shape of GNSs were visualized using an Osiris transmission electron microscope (TEM) at 200 keV. The morphology of complete immunocomplexes was visualized using a Zeiss Merlin scanning electron microscope (SEM). The Raman measurements were taken for 5 s exposure time using a Renishaw inVia Raman microscope system with a 785 nm laser that delivered ~30 mW of power. A ×50 objective lens was used to focus a laser spot on the surface of microfluidic device. An oxygen plasma cleaner was used to bind the patterned PDMS layer onto a clean microscope glass slide.

### 4.3 Preparation of antibody‐conjugated magnetic beads capture probes

To prepare the antibody‐conjugated magnetic beads, 6.65 μl of carboxylated magnetic beads were separated by a magnet and washed twice with 100 μl of 25 mM MES (pH 5) for 10 min on an inverter (18 rpm). The surfaces of magnetic beads were active through the reaction with 50 μl of 50 mg/ml EDC (dissolved in cold 25 mM MES) and 50 μl of 50 mg/ml NHS (dissolved in cold 25 mM MES) on an inverter (18 rpm) at room temperature for 30 min. Next, the magnetic beads were separated by a magnet and washed twice with 10X PBS (pH 7.4). Then, the magnetic beads were resuspended in 1 ml of 0.006 mg/ml anti‐cTnI or 0.41 ml of 0.006 mg/ml anti‐NPY antibodies (in 1X PBS) with gentle mixing (4 rpm) at 4°C. Nonspecifically bound antibodies were washed three times with 1X PBS (pH 7.4). Unreacted carboxylic groups were deactivated with 50 mM Tris (pH 7.4) with gentle mixing (4 rpm) at 4°C. The final magnetic beads were then washed three times with 1X PBS and stored in 1X PBS (pH 7.4) at 4°C for future use.

### 4.4 Preparation of functionalized gold nanostars detection probes

GNSs were synthesized through the one‐step and seedless method, as described in our previously published procedures.^19^ First, 18 ml of Milli‐Q water at 18 MΩ was mixed with 12 ml of 200 mM HEPES (pH 7.4 ± 0.2) by gentle inversion. Next, 300 μl of 20 mM chloroauric acid was added. The solution was mixed by gentle inversion and left undisturbed at room temperature for 75 min. To conjugate Raman tags to the GNS surfaces, 3 μl of 10 mM DTNB or pMBA (in 100% ethanol) was added to 30 ml of GNSs and reacted for 15 min with constant stirring at 4°C. The solution was then centrifuge at 6000 rpm for 20 min to remove excess Raman tags. GNS‐DTNB or GNS‐pMBA was resuspended with Milli‐Q water (18 MΩ) at a concentration of 1.14 mg/ml. Afterwards, 25 μl of 1 mg/ml of P2 or 10 μl of 1 mg/ml NP3 peptide was added to GNS‐DTNB or GNS‐pMBA, respectively, and reacted for 1 hr with gentle mixing (4 rpm) at 4°C. Lastly, the fully functionalized GNSs (GNS‐DTNB‐P2 or GNS‐pMBA‐NP3) were centrifuged at 4000 rpm and resuspended in Milli‐Q water (18 MΩ) at a concentration of 5.72 mg/ml. The solution was stored at 4°C for future use.

### 4.5 Singleplexed biomarker detection

The prepared antibody‐conjugated magnetic beads (50 μl) were added to a well of the passivated microfluidic device through the inlet channel. Afterwards, 50 μl of cTnI or NPY at various concentrations spiked with human serum was added into the well and allowed to mix with magnetic beads for 1 hr at 4°C. The cTnI concentrations studied here were 0.004, 0.007, 0.02, 0.05, 0.1, 0.5, 1, 10, 50, 200, 600, 1,000, 3,000, 5,000, 10,000, 16,000, and 28,000 ng/ml. The NPY concentrations studied were 0.08, 0.12, 0.3, 0.5, 1, 10, 100, 200, 600, 800, 1,500, 3,000, 4,000, 5,000, and 9,000 ng/ml. The well was washed three times with 1X PBS by flowing through the inlet channel and then collecting the waste with a syringe from the outlet channel. Then 50 μl of prepared GNS‐DTNB‐P2 or GNS‐pMBA‐NP3 was added to the well and allowed to mix for 1 hr at 4°C. The unbound GNSs were suctioned out by a syringe as waste, and the well was washed three times with Milli‐Q water (18 MΩ). The microfluidic device was dried for 10 min at room temperature and then imaged using a Renishaw inVia Raman microscope system. A blank sample was prepared in the absence of cTnI or NPY and was used as a control. Each sample was prepared with three replicates. At least 300 Raman spectra from different locations were obtained per replicate of sample.

### 4.6 Multiplexed biomarker detection

To assess the feasibility of multiplexed biomarker detection, 25 μl of anti‐cTnI‐conjugated magnetic beads and 25 μl of anti‐NPY‐conjugated magnetic beads were mixed and added to a well of the passivated microfluidic device through the inlet channel. cTnI (25 μl) and NPY (25 μl) at targeted concentrations spiked with human serum were added into the well and allowed to mix for 1 hr at 4°C. The combinations of biomarker concentration tested here were 3,000 ng/ml cTnI +1,500 ng/ml NPY, 1000 ng/ml cTnI +600 ng/ml NPY, 400 ng/ml cTnI +200 ng/ml NPY, 200 ng/ml cTnI +100 ng/ml NPY, 5 ng/ml cTnI +10 ng/ml NPY, 0.5 ng/ml cTnI +1 ng/ml NPY, and 0.05 ng/ml cTnI +0.3 ng/ml NPY. Note: these antigens were spiked with human serum. The well was washed three times with 1X PBS by flowing through the inlet channel and then collecting the waste with a syringe from the outlet channel. Then 20 μl of GNS‐DTNB‐P2 and 40 μl of GNS‐pMBA‐NP3 were added into the well and allowed to mix for 1 hr at 4°C. The unbound GNSs were suctioned out by a syringe as waste, and the well was washed three times with Milli‐Q water (18 MΩ). The microfluidic device was dried for 10 min at room temperature and then imaged using a Renishaw inVia Raman microscope system. A blank sample was prepared in the absence of cTnI and NPY and was used as a control. Each sample was prepared with three replicates. At least 300 Raman spectra were obtained per replicate of sample.

### 4.7 Reusability

The reusability of PRADA was leveraged with the magnetic beads, as removal of the magnet allowed us to wash off the entire immunoassay via gentle rinsing of the device. Note: the magnetic beads were not covalently attached to the glass surface but held in place with the magnet. The device was then cleaned with acetone followed by Milli‐Q water (18 MΩ). The Raman spectra for the cleaned device were then measured to ensure that there were no residual signals from the previous sample. The devices were also viewed in the microscope to ensure all the magnetic beads were washed off. The entire assay was repeated, 14 times with the same microfluidic device to demonstrate reusability.

### 4.8 Microfluidic device fabrication and passivation

All steps regarding the fabrication of microchannel patterns was performed using facilities within the cleanroom affiliated with the Vanderbilt Institute of Nanoscale Science and Engineering (VINSE). To make a microchannel mold, mr‐DWL_40 resist was cast on a clean silicon wafer and spin‐coated at 1000 rpm for 1 min, yielding a 60 μm‐thick resist layer. Then designed patterns were directly written into the photoresist using a laser writer (Heidelberg, μPG 101). The wafer with patterned resist was coated with a thin layer of TCPS to facilitate subsequent removal. To make a microfluidic device, liquid polydimethylsiloxane (PDMS) (Sylgard 184) was mixed in a 1:10 ratio of curing agent and PDMS resin, degassed in a desiccator and carefully poured onto the resist mold placed in a petri dish. After curing in an oven for 3 hr at 65°C, the PDMS layer was cut and peeled off the resist mold. Holes were punched at the inlet and outlet of the microchannels using a 8.5 mm internal diameter punch. A clean microscope glass slide was bonded to the patterned PDMS layer by exposing to an oxygen plasma for 4 min. Each well of the microfluidic device was then passivated with 150 μl of 20 mM mPEG‐silane (dissolved in 100% acetone) for 1 hr at room temperature to avoid nonspecific binding. The microfluidic device was cleaned with Milli‐Q water (18 MΩ) and dried with nitrogen. The passivated device was stored at −20°C in glovebox for future use.

### 4.9 Statistical analysis

The LOD for the assay was estimated as follows: LOD = LOB +1.645(SD_lowest concentration sample_), where LOB (limit of blank) was obtained by LOB = mean_blank_ + 1.645(SD_blank_), in which the average signal of the blank (the immunocomplex without antigen) is added to 5% false‐negative rate. All data are presented as mean ± standard deviation (SD). The sensitivity curves of both cTnI and NPY were fitted with four‐parameter logistic function using the GraphPad Prism8 program. Passing‐Bablok regression analysis was performed on patient samples using the MedCalc program. A custom MATLAB code was used to perform smoothing and biological fluorescent background subtraction of Raman spectra. Smoothing of the data were done by following the Savitzsky and Golay method with fifth order and coefficient value of 33. Modified polynomial fit method was performed to subtract the background fluorescence. A polynomial with seventh order was used to fit the Raman spectra with threshold of 0.0001.

## CONFLICT OF INTERESTS

The authors declare no competing interest.

## Supporting information


**Appendix** **S1**: Supporting Information
