# Supplementary material for: PRADA: Portable Reusable Accurate Diagnostics with nanostar Antennas for multiplexed biomarker screening
Source: Bioeng Transl Med. 2020 May 15;5(3):e10165. doi: 10.1002/btm2.10165 (PMC7510456; doi:10.1002/btm2.10165)
Supplement: Supplementary file 1 — Appendix S1: Supporting Information [file BTM2-5-e10165-s001.docx]

**Supporting Information**

**PRADA: *P*ortable *R*eusable *A*ccurate *D*iagnostics with nanostar *A*ntennas for Multiplexed Biomarker Screening**

Xiaona Wen^1^ | Yu-Chuan Ou^1^ | Holly F. Zarick^1^ | Xin Zhang^2^ | Anthony B. Hmelo^3^ | Quinton J. Victor^1^ | Eden P. Paul^1^ | Joseph M. Slocik^4^ | Rajesh R. Naik^4^ | Leon M. Bellan^2^ | Eugene C. Lin^5^ | Rizia Bardhan^*6,7^

^1^Department of Chemical and Biomolecular Engineering, Vanderbilt University, Nashville, Tennessee 37235, United States.

^2^Department of Mechanical Engineering, Vanderbilt University, Nashville, Tennessee 37235, United States.

^3^Department of Physics and Astronomy, Vanderbilt University, Nashville, Tennessee 37235, United States.

^4^Materials and Manufacturing Directorate and 711th Human Performance Wing, Air Force Research Laboratory, Wright-Patterson Air Force Base, Dayton, Ohio 45433, United States.

^5^Department of Chemistry and Biochemistry, National Chung Cheng University, Chiayi 62106, Taiwan.

^6^Department of Chemical and Biological Engineering, Iowa State University, Ames, Iowa 50012, United States.

^7^Nanovaccine Institute, Iowa State University, Ames, Iowa 50012, United States.

*Corresponding Author: [rbardhan@iastate.edu](mailto:rbardhan@iastate.edu)

**Table of Contents**

**p.S2: Figure S1.** Concentration-dependent SEM images.

**p.S2: Figure S2.** Raman and SEM micrographs of ideal and non-ideal conditions for PRADA.

**p.S3: Table S1.** Fitting parameters for the sensitivity curve of cTnI and NPY.

**p.S3: Table S2.** Calculations for limit of detection (LOD) of cTnI and NPY.

**p.S3: Figure S3.** Raman spectra of cTnI for 11 different patient samples using PRADA.

**p.S4: Table S3.** Passing-Bablok regression analysis of cTnI for 11 patient samples.

**p.S4: Table S4.** Comparison of analytical sensitivity parameters at 10% coefficient variation of the most recent cardiac troponin immunoassay.

**
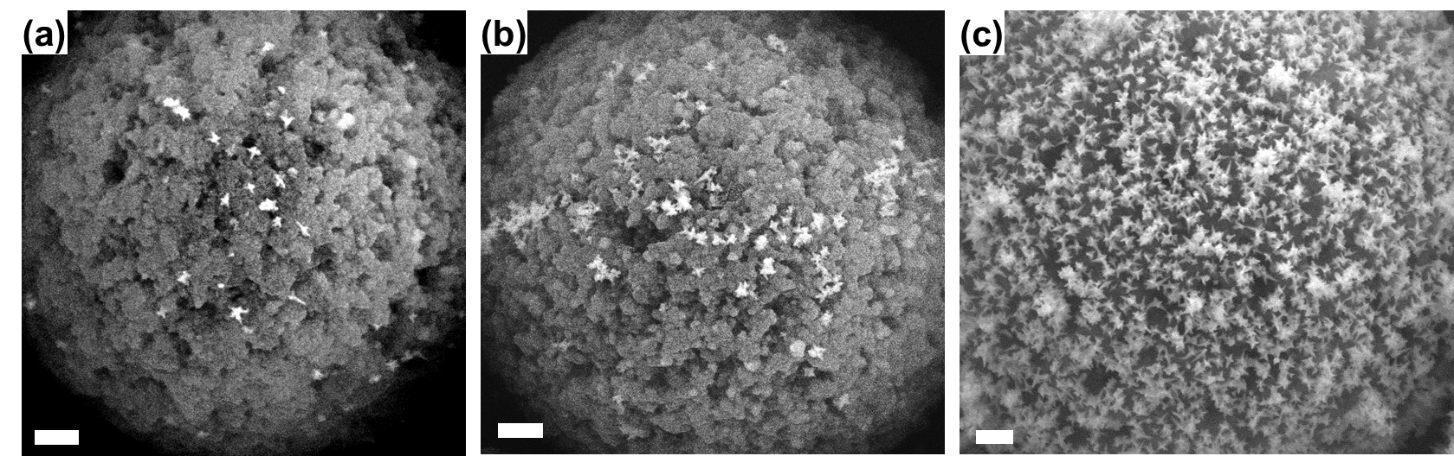
**

**Figure S1.** Concentration-dependent SEM micrographs showing the complete immunocomplexes with magnetic bead capture probes + antigens + peptide coated GNS-SERS barcode detection probes. Images are shown at (a) low (1 ng/mL), (b) medium (50 ng/mL), and (c) high (3000 ng/mL) concentration of cTnI. Scale bar is 200 nm in a – c.


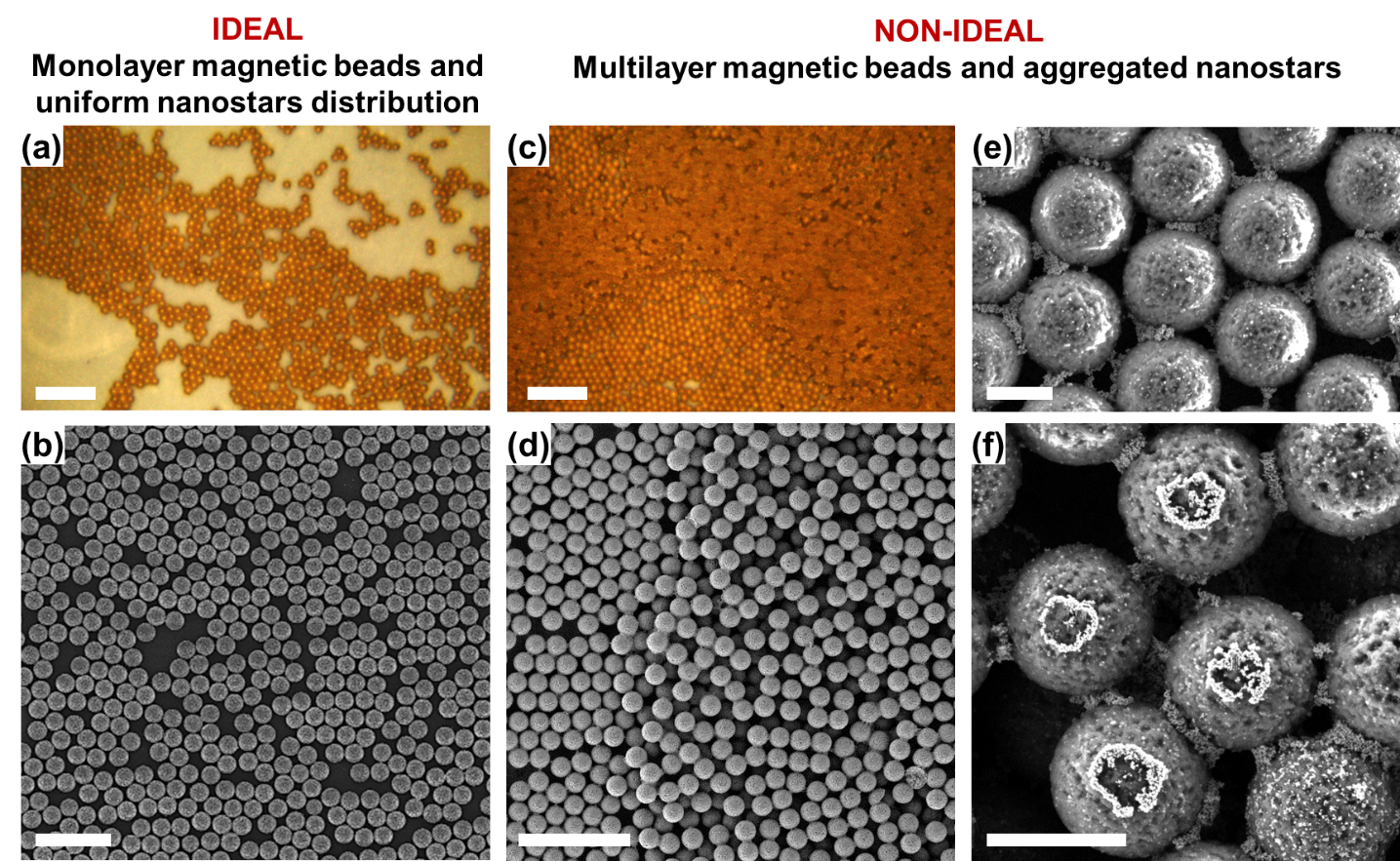


**Figure S2.** Raman images, and SEM micrographs to showcase the (a,b) ideal conditions and (c-f) non-ideal conditions to perform PRADA. (a,b) PRADA was optimized to consistently generate ideal conditions with monolayers of magnetic beads and uniform distribution of nanostars resulting in highly reproducible results (also see Figure 2c-e). SERS measurements were not performed if prepared samples were non-ideal to minimize hot-spots and irreproducible results. Non-ideal conditions include (c,d) multilayers of magnetic beads and (e,f) non-uniform aggregated nanostars on beads. Scale bar is 20 µm in (a) and (c), 10 µm in (b) and (d), and 1 µm in (e) and (f).

**Table S1.** Fitting parameters for the sensitivity curve of cTnI and NPY.

|  | **cTnI** | **NPY** |
| --- | --- | --- |
| x_0_ | 5.495 × 10^24^ | 2.884 × 10^16^ |
| y_0_ | 402.7 | 861.5 |
| a | 1.766 × 10^6^ | 2.483 × 10^6^ |
| b | 0.122 | 0.222 |
| R^2^ | 0.995 | 0.996 |

The sensitivity curve of cTnI and NPY in the quantification region was fitted using the 4PL function given below.

$$y=y_{0}+\frac{a}{1+\left( \frac{x_{0}}{x} \right)^{b}}$$

**Table S2.** Calculations for limit of detection (LOD) of cTnI and NPY.

|  | **cTnI** | **NPY** |
| --- | --- | --- |
| Mean_blank_ | 796.82 | 766.29 |
| SD_blank_ | 95.77 | 97.72 |
| LOB = Mean_blank_ + 1.645 (SD_blank_) | 954.37 | 927.05 |
| SD_lowest concentration sample_ | 224.04 | 171.42 |
| LOD (Intensity, cps) = LOB + 1.645 (SD_lowest concentration sample_) | 1322.92 | 1209.02 |
| LOD (Concentration, ng/mL) | 0.0055 | 0.12 |


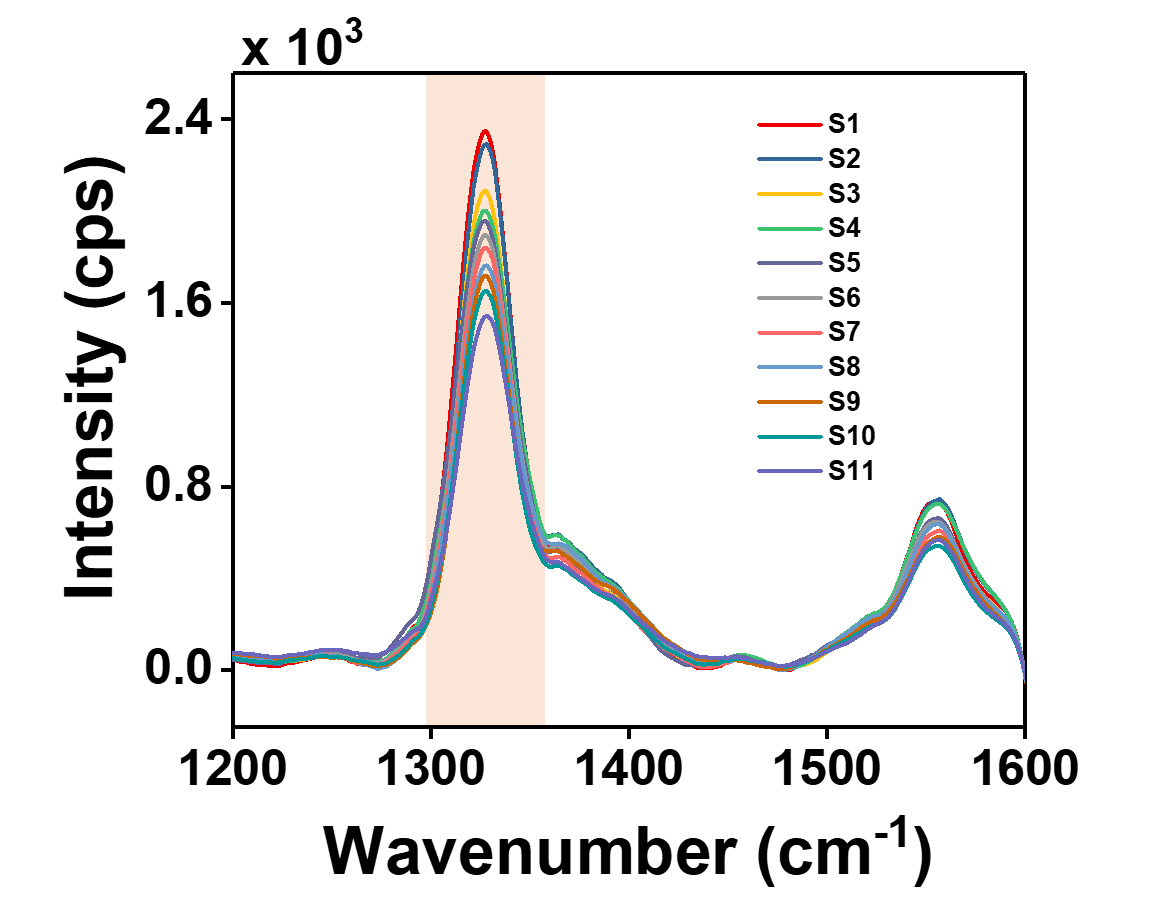


**Figure S3.** SERS spectra of cTnI detection for 11 different patient samples using PRADA. All other patient information is shown in Figure 5a.

**Table S3.** Passing-Bablok regression analysis of cTnI detection for the 11 patient samples.

| **Regression equation** | y = -0.01134 + 0.9601 × x |
| --- | --- |
| **Systematic differences** | |
| Intercept A | -0.01134 |
| 95% CI | -0.02534 to 0.01475 |
| **Proportional differences** | |
| Slope B | 0.9601 |
| 95% CI | 0.8850 to 1.0777 |
| **Linear model validity** | No significant deviation from linearity (P = 1.00) |

**Table S4.** Comparison of analytical sensitivity parameters at 10% coefficient variation of PRADA and other cardiac troponin immunoassays recently published in the literature, and commercially available. SPR: Surface Plasmon Resonance, LSPR: Localized Surface Plasmon Resonance, SERS: Surface Enhanced Raman Spectroscopy, CV: Cyclic voltammetry, EIS: electrochemical impedance spectroscopy, ELISA: Enzyme-linked Immunosorbent Assay.

| **Platform** | **LOD (ng/mL)** | **LOQ (ng/mL)** | **Multiplexing** | **Ref./Manufacturer** |
| --- | --- | --- | --- | --- |
| SPR | 0.50 | N/A | No | Ref.^1^ |
| LSPR | 0.035 | N/A | No | Ref.^2^ |
| SERS | 0.034 | N/A | No | Ref.^3^ |
| CV/EIS | 0.024 | N/A | No | Ref.^4^ |
| CV | 0.024 | N/A | No | Ref.^5^ |
| **PRADA** | **0.0055** | **0.032** | **Yes** | **This work** |
| ELISA | 0.1 | N/A | No | ThermoFisher^6^ |
| Luminex | 0.069 | N/A | Yes | MilliporeSigma^7^ |
| ARCHITECT STAT | 0.010 | 0.050 | No | Abbott^8^ |
| ELECSYS | 0.0050 | 0.012 | No | Roche^9^ |
| ADVIA Centaur | 0.0022 | 0.0027 | No | Siemens^10^ |
| AIA | 0.0021 | 0.031 | No | Tosoh Bioscience^11^ |

**References**

1. Pawula M, Altintas Z, Tothill IE. SPR detection of cardiac troponin T for acute myocardial infarction. *Talanta.* 2016;146;823-830.

2. Tadepalli S, Kuang Z, Jiang Q, et al. Peptide functionalized gold nanorods for the sensitive detection of a cardiac biomarker using plasmonic paper devices. *Sci. Rep.* 2015;5;16206.

3. Chon H, Lee S, Yoon S-Y, et al. SERS-based competitive immunoassay of troponin I and CK-MB markers for early diagnosis of acute myocardial infarction. *Chem. Commun.* 2014;50;1058-1060.

4. Jo H, Her J, Lee H, et al. Highly sensitive amperometric detection of cardiac troponin I using sandwich aptamers and screen-printed carbon electrodes. *Talanta.* 2017;165;442-448.

5. Lee T, Lee Y, Park SY, et al. Fabrication of electrochemical biosensor composed of multi-functional DNA structure/Au nanospike on micro-gap/PCB system for detecting troponin I in human serum. *Colloids Surf. B. Biointerfaces.* 2019;175;343-350.

6. ThermoFisher. (2020). Cardiac troponin I (TNNI3) human ELISA kit. Retrieved from <https://www.thermofisher.com/elisa/product/Cardiac-Troponin-I-TNNI3-Human-ELISA-Kit/EHTNNI3>

7. MilliporeSigma. (2020). MILLIPLEX MAP human cardiovascular disease (CVD) magnetic bead panel 1 - cardiovascular disease multiplex assay. Retrieved from [https://www.emdmillipore.com/US/en/product/MILLIPLEX-MAP-Human-Cardiovascular-Disease-CVD-Magnetic-Bead-Panel-1-Cardiovascular-Disease-Multiplex-Assay,MM_NF-HCVD1MAG-67K?CatalogCategoryID=#](https://www.emdmillipore.com/US/en/product/MILLIPLEX-MAP-Human-Cardiovascular-Disease-CVD-Magnetic-Bead-Panel-1-Cardiovascular-Disease-Multiplex-Assay,MM_NF-HCVD1MAG-67K?CatalogCategoryID=)

8. Shah ASV, Griffiths M, Lee KK, et al. High sensitivity cardiac troponin and the under-diagnosis of myocardial infarction in women: prospective cohort study. *BMJ*, 2015;350;g7873.

9. Koerbin G, Tate JR, Hickman PE. Analytical characteristics of the Roche highly sensitive troponin T assay and its application to a cardio-healthy population. *Ann. Clin. Biochem.* 2010;47;524-528.

10. Payne R, Halik L, Ma J, et al. Performance evaluation of the Siemens ADVIA centaur high sensitivity troponin I assay. *Eur. Heart J.* 2017;38(suppl_1).

11. Masotti S, Musetti V, Prontera C, et al. Evaluation of analytical performance of a chemiluminescence enzyme immunoassay (CLEIA) for cTnI using the automated AIA-CL2400 platform. *Clin. Chem. Lab. Med.* 2018;56;e174.
